# Concentration–Polarization Electroosmosis near Insulating Constrictions within Microfluidic Channels

**DOI:** 10.1021/acs.analchem.1c02849

**Published:** 2021-10-27

**Authors:** Raúl Fernández-Mateo, Víctor Calero, Hywel Morgan, Antonio Ramos, Pablo García-Sánchez

**Affiliations:** † School of Electronics and Computer Science, University of Southampton, Southampton SO17 1BJ, United Kingdom; ‡ Departamento de Electrónica y Electromagnetismo, Facultad de Física, Universidad de Sevilla, Avda. Reina Mercedes s/n, 41012 Sevilla, Spain

## Abstract

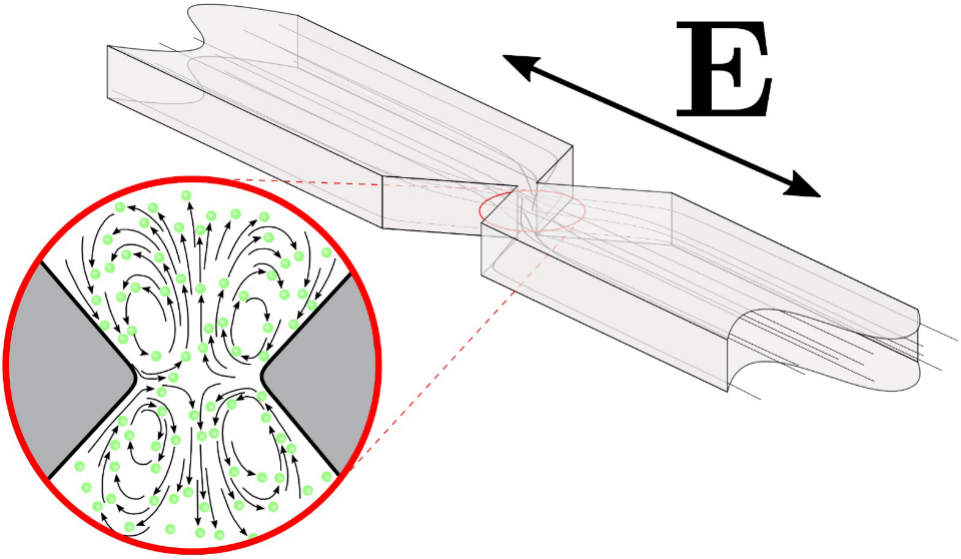

Electric fields are commonly used to trap and separate micro- and nanoparticles near channel constrictions in microfluidic devices. The trapping mechanism is attributed to the electrical forces arising from the nonhomogeneous electric field caused by the constrictions, and the phenomenon is known as insulator-based-dielectrophoresis (iDEP). In this paper, we describe stationary electroosmotic flows of electrolytes around insulating constrictions induced by low frequency AC electric fields (below 10 kHz). Experimental characterization of the flows is described for two different channel heights (50 and 10 μm), together with numerical simulations based on an electrokinetic model that considers the modification of the local ionic concentration due to surface conductance on charged insulating walls. We term this phenomenon concentration–polarization electroosmosis (CPEO). The observed flow characteristics are in qualitative agreement with the predictions of this model. However, for shallow channels (10 μm), trapping of the particles on both sides of the constrictions is also observed. This particle and fluid behavior could play a major role in iDEP and could be easily misinterpreted as a dielectrophoretic force.

Electric fields have been widely used to manipulate small particles dispersed in aqueous solutions.^1,2^ Many research groups have demonstrated electric-field induced trapping of particles and molecules within constrictions in microfluidic channels.^3^ For example, early work by Chou et al.^4^ showed that DNA could be trapped and enriched between insulating obstacles fabricated in a quartz wafer. Electrical manipulation and trapping of latex colloids within arrays of glass posts was demonstrated by Cummings and Sigh.^5^ Liao et al.^6^ used nanoconstrictions and a combination of AC and DC fields for protein enrichment in physiological media. Lapizco-Encinas et al. reported concentration and separation of live and dead bacteria^7^ and concentration of proteins in low conductivity electrolytes using DC fields in an array of cylindrical insulating posts etched in glass.^8^ Physher and Hayes demonstrated separation of bacteria populations using a series of constrictions with decreasing width along a channel subject to a DC field.^9^

All these results are based on the application of an electric field along constrictions and/or obstacles in a channel where the electric current is squeezed, giving rise to a spatially nonuniform electric field. In this situation, when a polarizable particle is in the presence of a nonhomogeneous electric field, a net electrical force is exerted on it and the resulting particle motion is known as dielectrophoresis (DEP).^10,11^ Because the field distortion is created by insulating objects, the technique is called insulating-DEP (iDEP) or electrodeless-DEP (eDEP), although the term eDEP is more commonly used to denote electrode-based DEP.

In this paper we study fluid flows generated in the vicinity of insulating constrictions due to the presence of a low frequency (<10 kHz) AC electric field, similar to those used in iDEP. The study is motivated by recent observations of quadrupolar fluid flow induced by AC fields around insulating micropillars^12,13^ and charged dielectric microspheres.^14^ The constriction consists of a simple triangular shaped insulator within a long microchannel with a square cross-section, similar to the geometry used for particle trapping in the work of Chou et al.^4^ and Su et al.^15^ The fluid flow profile is measured using 500 nm diameter tracer particles. Figure 1(a) shows a diagram of the channel and the constriction, together with the inlet and outlet reservoirs within which electrodes are placed. The flow characteristics are described as a function of different experimental parameters (frequency, amplitude, and electrolyte conductivity). We also demonstrate that the extent and influence of the fluid rolls depends on the height of the channel.

**Figure 1:**
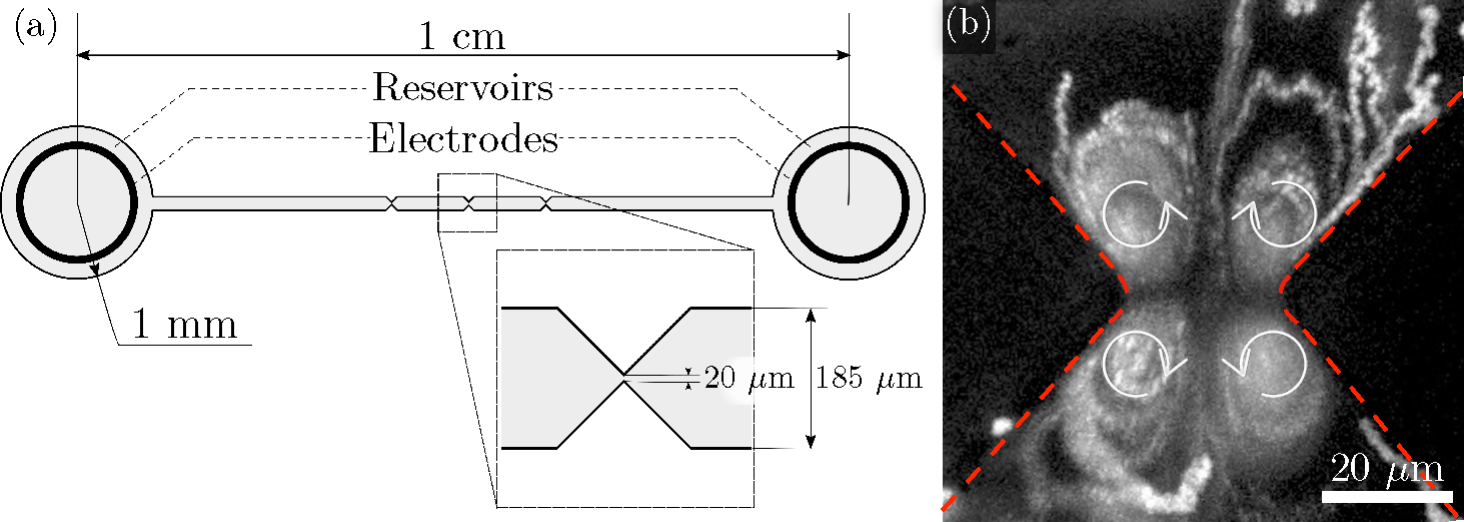
(a) Diagram of the microfluidic channel showing three constrictions (top view). A voltage is applied using metal needle electrodes inserted into the reservoirs. Two channel heights were used: 10 and 50 μm. (b) An image demonstrating an example of quadrupolar fluid vortices observed around a constriction.

Experimental measurements of the fluid velocity are in qualitative agreement with predictions of our recent theory of concentration–polarization electroosmosis (CPEO).^13^ Stationary flow vortices induced by AC fields, similar to those shown in Figure 1(b), arise from gradients in electrolyte concentration caused by the surface conductance on the charged walls of insulating objects such as glass or polydimethylsiloxane (PDMS). The flow patterns are seen in relatively low conductivity electrolytes; they are not electrothermal in origin^16^ although such flows may occur in higher conductivity electrolytes.^17^ In addition to being of fundamental interest, these flows are likely to influence the behavior of iDEP devices and provide further insights into the operation and application of techniques such as iDEP and electrokinetic deterministic lateral displacement (DLD) where AC fields modify particle behavior.^18−21^

## Experimental Section

### Experimental Setup and Methods

The microfluidic devices (Figure 1) were made of PDMS using standard soft lithography. The constrictions are 20 μm wide, and channels with two different heights were made: 50 and 10 μm. Aqueous solutions of KCl with conductivities σ = {1.7, 6.1, 12.2} mS/m and pH approximately 5.5 were seeded with polystyrene fluorescent nanoparticles (500 nm diameter, zeta-potential in KCl 6.6 mS/m is ζ=–63 ± 6 mV) which act as tracers to map the fluid flow. These were imaged with a fluorescence microscope with a 100× objective. Prior to experiments, the PDMS channels were primed with a solution of 0.1% (w/v) Pluronic F-127, which is a nonionic surfactanct that adsorbs onto the PDMS walls and minimizes adhesion of the tracer particles. AC voltages of an amplitude up to 1600 V peak-to-peak were applied along the channel with two metal needles placed 1 cm apart at the inlet and outlet of the channel. Videos of the tracer particles were analyzed with “PIV lab”, a software for particle image velocimetry (PIV).^22^ The liquid in the channel was renewed after each measurement to minimize any changes in electrical properties caused by Faradaic reactions due to the low-frequency field. A pressure controller (Elveflow OB1MK3+) was used to refresh the liquid in the channel and to stop the flow for the measurements.

### Experimental Results with Tall Channels

**Figure 2:**
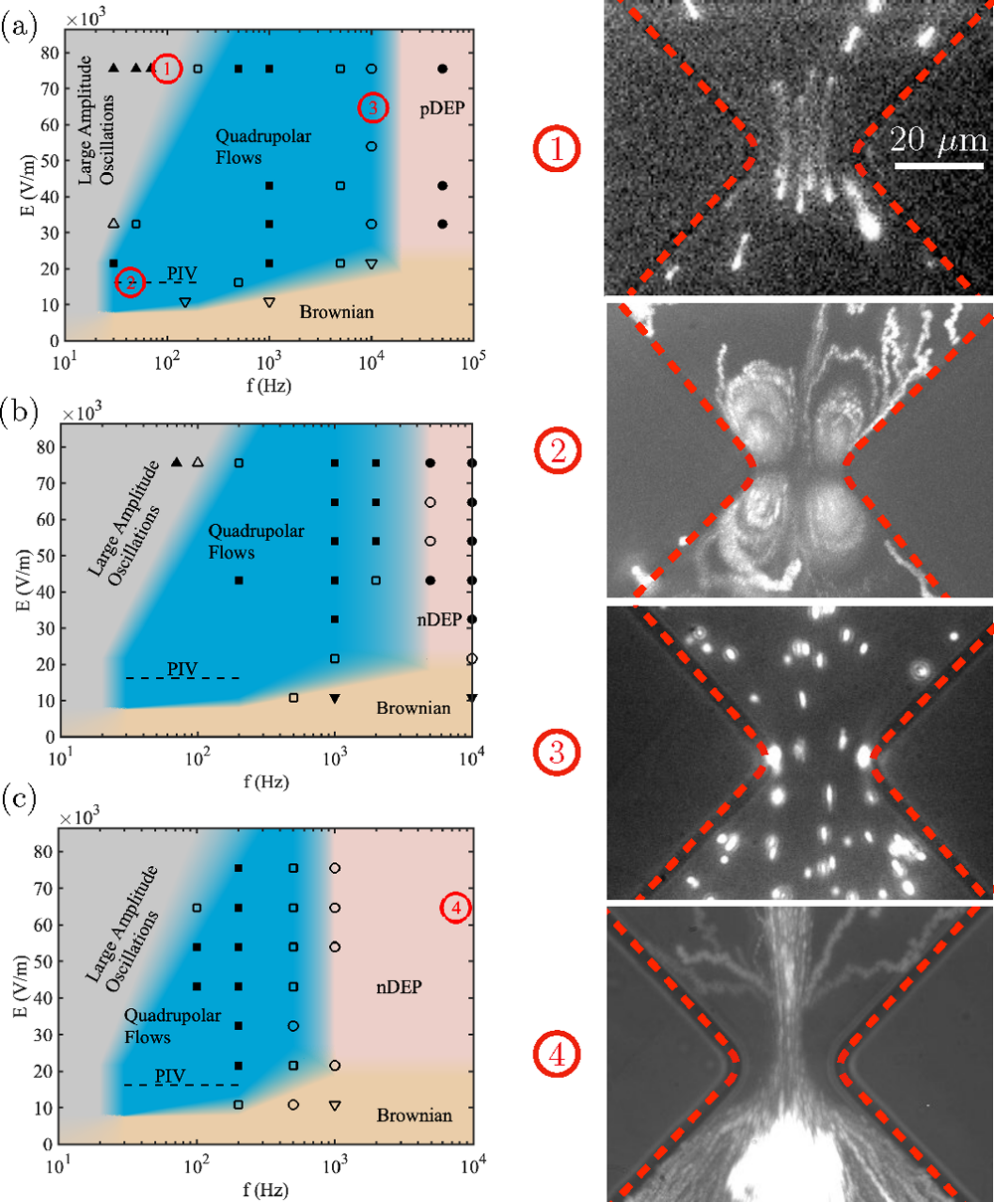
Maps showing the general behavior of the colloidal particles (500 nm diameter) near a channel constriction as a function of amplitude and frequency of the AC electric field. Three different conductivities of electrolyte were used, and a map is shown for each of these: (a) 1.7 mS/m, (b) 6.1 mS/m, (c) 12.2 mS/m. The dashed lines for each electrolyte conductivity indicate the frequency range within which fluid velocities were measured by PIV. The channels are 50 μm tall, and the constriction is 20 μm wide. The experimental points used to construct the maps are shown, and the predominant behavior is highlighted as (■) quadrupolar flows, (●) DEP, (▲) large amplitude oscillations, (▼) Brownian motion. Solid symbols indicate that a single behavior dominated. Open symbols indicate a mix of behaviors.

Figure 2 shows a set of diagrams describing the behavior of the fluorescent tracer beads near the constriction in a 50  μm tall channel as a function of the electric field amplitude **E** and frequency *f*. In these plots, **E** is the amplitude of the electric field far from the constriction. The figure has a diagram for each electrolyte conductivity, and the symbols indicate the points on the map where experimental observations were recorded.

**Figure 3:**
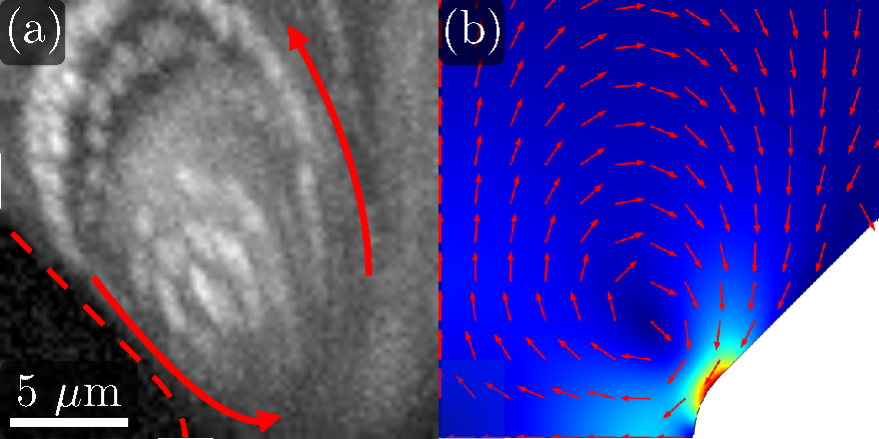
(a) Experimental streamlines in the constriction for KCl with a conductivity of 1.7 mS/m, an applied field amplitude of 15 kV/m, and frequency of 65 Hz. (b) COMSOL simulations reproducing the experimental system. The surface plot depicts the solution of the velocity field magnitude, while the arrow plot corresponds to the fluid velocity direction.

The maps show that particle electrophoresis dominates at low frequencies, manifesting as an oscillatory motion that drives the particle from one side of the constriction to the other and back (see image in Figure 2(a)). The amplitude of the oscillating electrophoresis decreases with increasing frequency and eventually vanishes for frequencies larger than tens of Hertz depending on the amplitude of the electric field. In this situation, when the electrophoresis becomes relatively small, four steady flow vortices were observed at the microfluidic constriction. This regime corresponds to the blue regions in the maps of Figure 2, and example images of the quadrupolar flows are shown in the figure. These images were obtained by superimposing several video frames of particle motion. Figure 3(a) shows a larger image of one of these rolls. As discussed below, we hypothesize that concentration polarization drives these quadrupolar flows which we refer to as concentration–polarization electroosmosis (CPEO).^13^

Further increasing the frequency of the AC signal leads to a decrease in the velocity of the flow vortices. For the lowest electrolyte conductivity (1.7 mS/m), the fluorescent beads accumulate at the tip of the triangular constrictions at frequencies of approximately 20 kHz and above. This frequency effectively establishes the boundary or transition between the quadrupolar flows and positive dielectrophoresis (pDEP) of the particles. This transition frequency is marked on Figure 2(a). At high frequencies, pDEP drives particle accumulation to regions of maximum field gradient at the tip of the constriction. To determine the transition frequency, the field was applied and the particle behavior was observed after approximately 2 min to allow the beads to accumulate at the tip of the constriction. Neither particle motion nor accumulation were observed for low values of electric field amplitude (≈10 kV/m and below). This is labeled on the map as “Brownian”. The absolute limits of this region are somehow arbitrary because they depend on experimental factors including the time-window of observation. Furthermore, the boundaries between the different regimes (Figures 2) will vary depending on particle size. For example, the DEP force varies with particle volume and is expected to dominate over a wider frequency range for larger particles.

Increasing the electrolyte conductivity to 6.1 mS/m reduces the transition frequency to 5 kHz (Figure 2(b)), while for a higher electrolyte conductivity of 12.2 mS/m, the transition frequency is around 1 kHz (Figure 2(c)). However, at these higher conductivities, the beads do not accumulate at the tip of the constriction but are expelled from the vicinity of the tip, as expected if the particles undergo negative dielectrophoresis (nDEP). This change from positive to negative DEP can be understood if the particle effective conductivity is evaluated from the O’Konski model^1^ as σ_p_ = 2*K*_s_/*a*, where *K*_s_ is the surface conductance of the particle. A typical value for *K*_s_ of latex beads is 0.5 nS^23^ and σ_p_ = 4 mS/m. This is in accordance with the observation of pDEP for 1.7 mS/m and nDEP for the two other conductivities.

**Figure 4:**
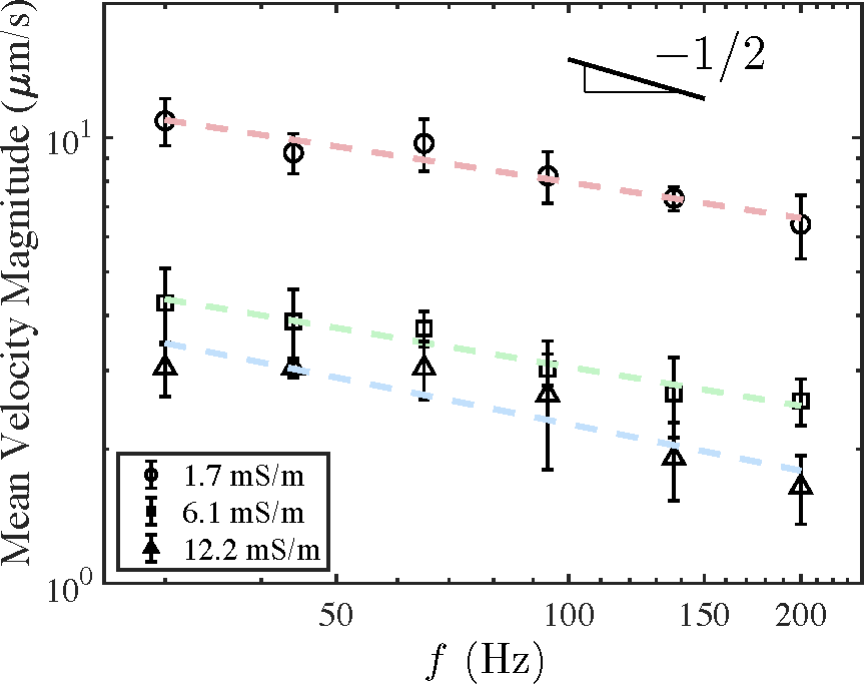
Results of PIV measurements for the average velocity magnitude as a function of frequency of the applied electric field. The amplitude of the applied field was 15 kV/m. Two trends can be clearly observed: the decay of the velocity magnitude with frequency (*f*^–1/2^ trend line shown) and with electrolyte conductivity.

Quantitative characterization of the quadrupolar flows was performed by measuring the average magnitude of the fluid velocity using PIV analysis in an area near the constriction containing a single vortex (see Figure 7 for an example area). Each of the four symmetric vortices were analyzed independently, and the mean magnitude of the velocity field was averaged across all four. The peak-to-peak voltage amplitude was kept constant at 300 V, corresponding to a field amplitude of 15 kV/m. The position in the map of these detailed measurements within the quadrupolar flow is indicated with the dashed lines in Figure 2. The data in Figure 4 gives the mean velocity as a function of frequency for each electrolyte conductivity. This velocity approximately decreases as , in agreement with our previous findings for the trend in CPEO velocity around a micropillar.^13^

### Experimental Results with Shallow Channels

**Figure 5:**
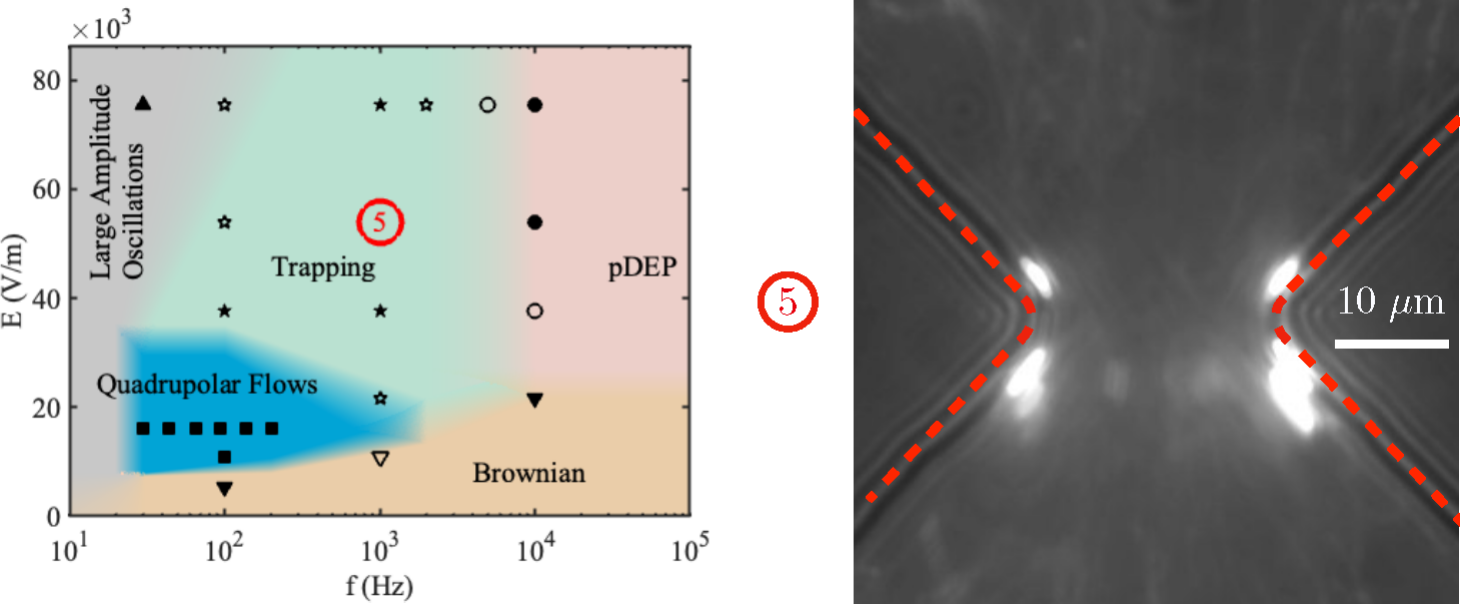
Particle behavior in shallow (10 μm) channels for a conductivity of σ = 1.7 mS/m. A trapping region emerges, where particles are concentrated at the sides of the constriction tips. This is shown in the image on the right, obtained by averaging the intensity of single image frames from a video over 2 min. Experimental points used to construct the maps are included to highlight the dominant behavior: (■) Quadrupolar flows, (★) trapping, (●) DEP, (▲) large amplitude oscillations, (▼) Brownian motion. Filled symbols indicate a single behavior dominated; open symbols indicate a mixture of behaviors.

Many iDEP devices use constrictions within microchannels that are shallower than those used in the previous section, typically around 10 μm tall or less.^4,5^ Therefore, the flow patterns were also measured for constrictions in channels with a reduced height of 10  μm. As for the taller channels, large-amplitude oscillations of the fluorescent beads were observed at low AC frequencies. Quadrupolar flows were also observed, but they only extended a short distance from the constrictions walls and dominated within a much smaller region of the map, namely for electric fields smaller than 30 kV/m and a frequency range from 30 Hz to 1 kHz (see blue region in Figure 5). For sufficiently high electric field magnitude and frequency (green region in Figure 5), beads were observed to accumulate on both sides of the constriction tips (see image in Figure 5). A similar behavior was also found around insulating pillars subjected to AC fields in a shallow channel (8 μm).^20^ This trapping is notably different from DEP trapping because the particles accumulate in different positions than those expected from nDEP or pDEP. Significantly, the trapping occurs in the same positions regardless of whether the particles experience pDEP or nDEP at higher frequencies. This phenomenon disappears for increasing frequency, and classical DEP behavior is observed if the magnitude of the electric field is large enough to overcome Brownian motion and diffusion. At present the theoretical basis for this “trapping” regime is not clear, but it is likely to play an important role in the behavior of iDEP devices.

### Experimental Results with Larger Particles

**Figure 6:**
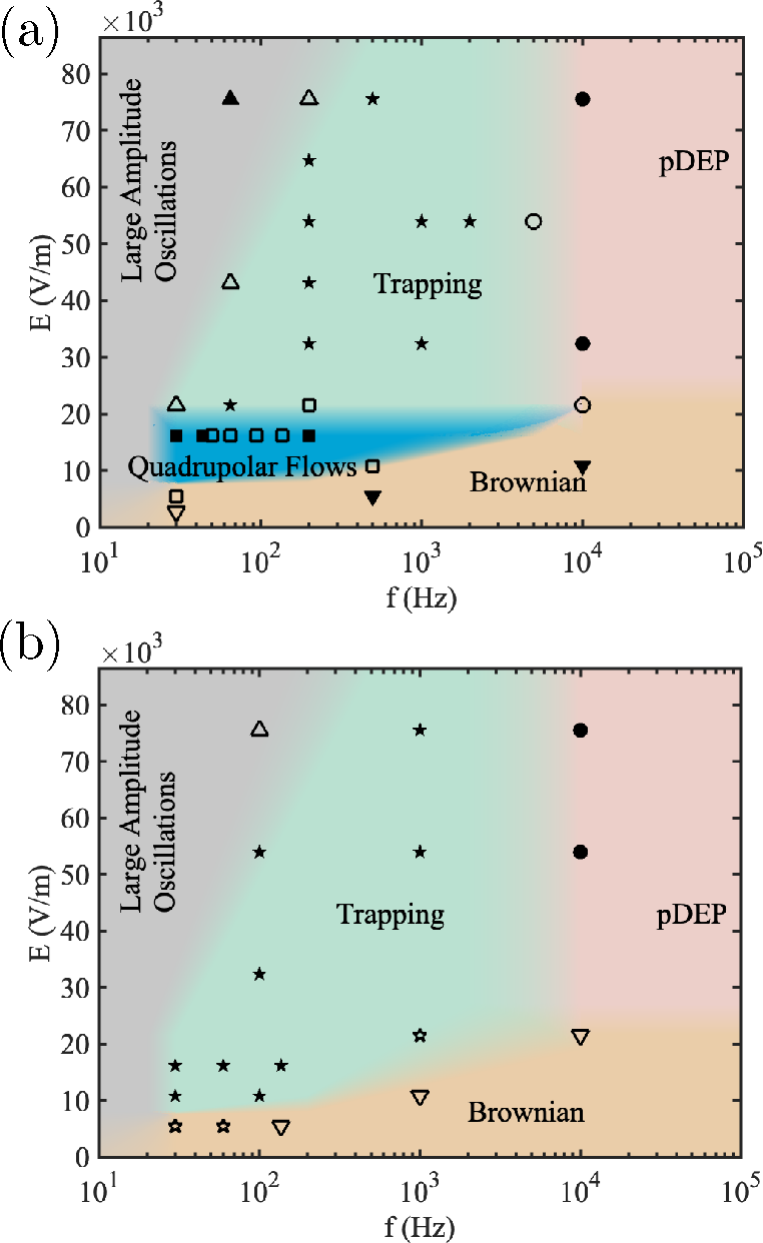
Behavior of 1 μm particles suspended in 1.7 mS/m KCl. (a) 50 μm tall channel and (b) 10 μm tall channel. (■) Quadrupolar flows, (★) trapping, (●) DEP, (▲) large amplitude oscillations, (▼) Brownian motion. Filled symbols indicate a single behavior dominated; open symbols indicate a mixture of behaviors.

To check the influence of particle size on fluid flow traceability, some experiments were performed using 1 μm diameter fluorescent particles (zeta-potential in KCl 6.6 mS/m is ζ = −71 ± 4 mV). Figure 6(a) shows the map for these particles in the tall channels and a conductivity of 1.7 mS/m. The following differences with respect to the 500 nm particles (Figure 2(a)) are found: (i) Positive DEP appears at slightly lower frequencies (10 kHz for 1 μm particles whereas for the 500 nm particles this frequency was 20 kHz), and (ii) most of the region of quadrupolar flows for the 500 nm particles is now occupied by the trapping region.

Figure 6(b) shows the map for the 1 μm particles in the shallow channel at a conductivity of 1.7 mS/m. Comparing this with the 500 nm particle map (Figure 5) shows that quadrupolar flows are not observed. Instead, trapping is observed in that region of the map.

The 1 μm particles were also used to measure the fluid velocity within the region of quadrupolar flows in Figure 6(a). The measured velocities are very close to the previous results with 500 nm particles (Figure 4). This confirms that the larger particles can be used as fluid flow tracers. In summary, the maps depend on particle size, but the 500 nm tracers can be used to trace and measure the flows over a wider range of velocities.

## Theoretical Analysis of the Quadrupolar Flows

Electroosmosis refers to the fluid motion induced by an electric field acting on the diffuse electrical layer of electrolytes close to the surface of a charged solid.^24,25^ This motion is commonly described via an effective slip velocity tangential to the solid wall, **u**_slip_, given by the Helmholtz–Smoluchowski formula:^24^
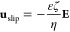
1where ε and η are, respectively, the electrolyte permittivity and viscosity. **E** is the amplitude of the applied electric field, and ζ (zeta-potential) is commonly defined as the electrical potential at the slip plane with the bulk solution.^26^

Equation 1 predicts an oscillatory slip velocity with a zero time-average value for the case of AC electric fields. Therefore, electroosmosis cannot account for our recent observations of a nonzero time-average electroosmostic velocity around dielectric microposts^12^ and corners^27^ in the presence of an AC field. In a recent paper,^13^ we showed that these flows can be explained by a model that considers the polarization of a modified electrolyte concentration (i.e., concentration polarization) that occurs due to surface conductance on the charged surface.^25^ Thus, we refer to this phenomenon as concentration–polarization electroosmosis (CPEO). In this section we compare the predictions of this theory with the experimental data presented in the previous section.

**Figure 7:**
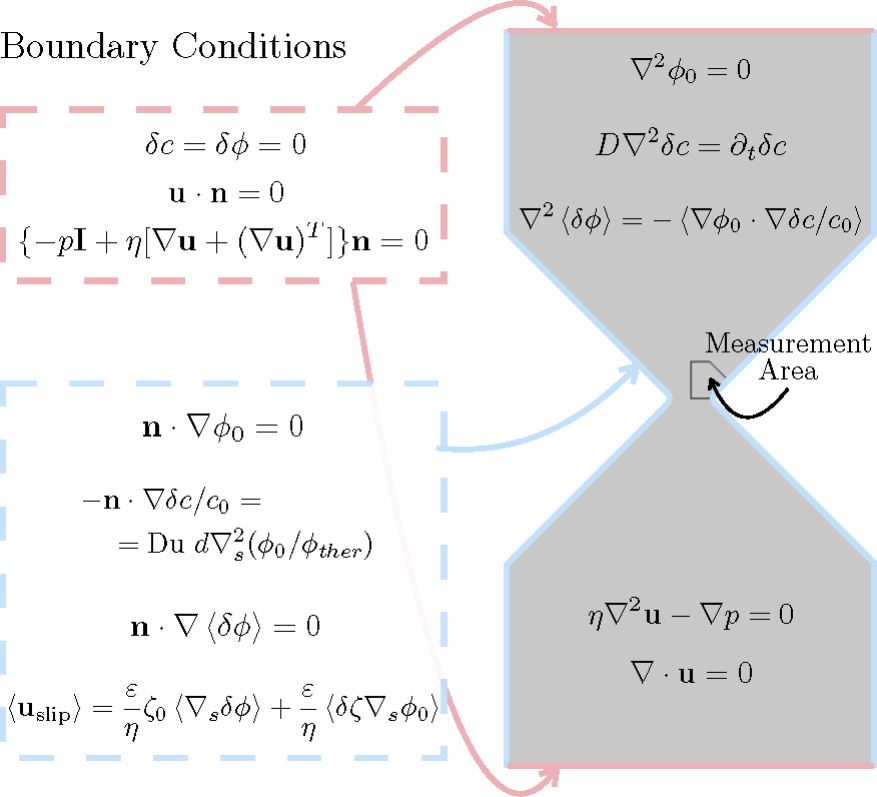
Summary of equations and boundary conditions for the electric potential, electrolyte concentration, and fluid velocity.

Our theoretical analysis^13^ follows the works of Schnitzer and Yariv^28,29^ that considered the electrophoresis of charged particles immersed in a symmetrical electrolyte. We extended the analysis to the case of AC signals and performed a linear expansion of the governing equations for a small Dukhin number (Du), the ratio of surface to bulk conductance.^25^ In this approximation, the electrical potential can be written as ϕ = ϕ_0_ + *δϕ*, where ϕ_0_ is the potential within the electrolyte for Du = 0, and *δϕ* is the perturbation as a consequence of surface conductance. In the present case, the electrolyte is subject to an AC field with magnitude *E*_0_ and angular frequency ω. Thus, ϕ_0_ in the electrolyte can be written as , where ϕ̃_0_ is the potential phasor and  indicates the real part of the argument between the brackets. The phasor ϕ_0_ is found by solving Laplace’s equation with boundary conditions of zero normal derivative on the channel walls (see Figure 7).

The electrolyte concentration is also written as *c* = *c*_0_ + *δc*, where *c*_0_ is the bulk concentration and *δc* is the perturbation due to the applied field. Neglecting advection, *c* satisfies the diffusion equation which implies that the phasor *δc̃* is a solution of *D*∇^2^*δc̃* = *iωδc̃*, where *D* is the diffusion coefficient of the ions in the electrolyte. We calculate *δc̃* in the domain of Figure 7 with the following boundary condition on the walls:
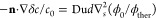
2where **n** is a unit vector normal to the wall and *d* is the characteristic length scale of the problem (constriction width in our case). ∇_s_^2^ is the Laplacian operator tangential to the wall surface. ϕ_ther_ = *k*_B_*T*/*e* ≈ 25 mV and used as the scale for electric potential. The Dukhin number, Du, is the ratio of surface to bulk conductance (Du = *K*_s_/*σd*, with *K*_s_ the surface conductance and σ the electrolyte conductivity). Equation 2 was derived^29^ for the case of thin diffuse layers. It assumes that the surface current is only due to counterions (co-ions are expelled from the diffuse layer), and it describes the balance of this current with the flux of ions coming from the bulk electrolyte (see also our previous paper^13^).

Changes in local concentration *δc* near the walls result in variations of the zeta-potential *δζ*. Using the Gouy–Chapman relation,^24^*δζ*/ϕ_ther_ = −*δc* tanh(ζ_0_/2ϕ_ther_)/*c*_0_. For the case of an AC voltage, *δζ* is also an oscillating function with the same angular frequency ω. From eq 1 it is found that this oscillating zeta-potential gives rise to a net time averaged electroosmotic velocity given by
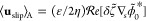
3where * indicates complex conjugate.

As shown in our previous work,^13^ gradients in electrolyte concentration lead to a rectified (nonzero time-average) electric field. This can be seen from the equation of current conservation for a symmetrical electrolyte that yields the following equation for the time-averaged component of the perturbation of the electrical potential:
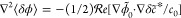
4

This rectified electric field acts on the charges in the intrinsic diffuse layer of the channel walls and generates a nonzero time average electroosmotic velocity given by
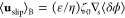
5

From the solutions of *δϕ* and *δc*, eqs 3 and 5 are evaluated on the channel walls and used as boundary conditions to determine the time-averaged fluid velocity (**u**) within the channel, which satisfies the Stokes equation:
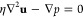
6
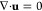
7

## Numerical Results and Comparison with Experiments

**Figure 8:**
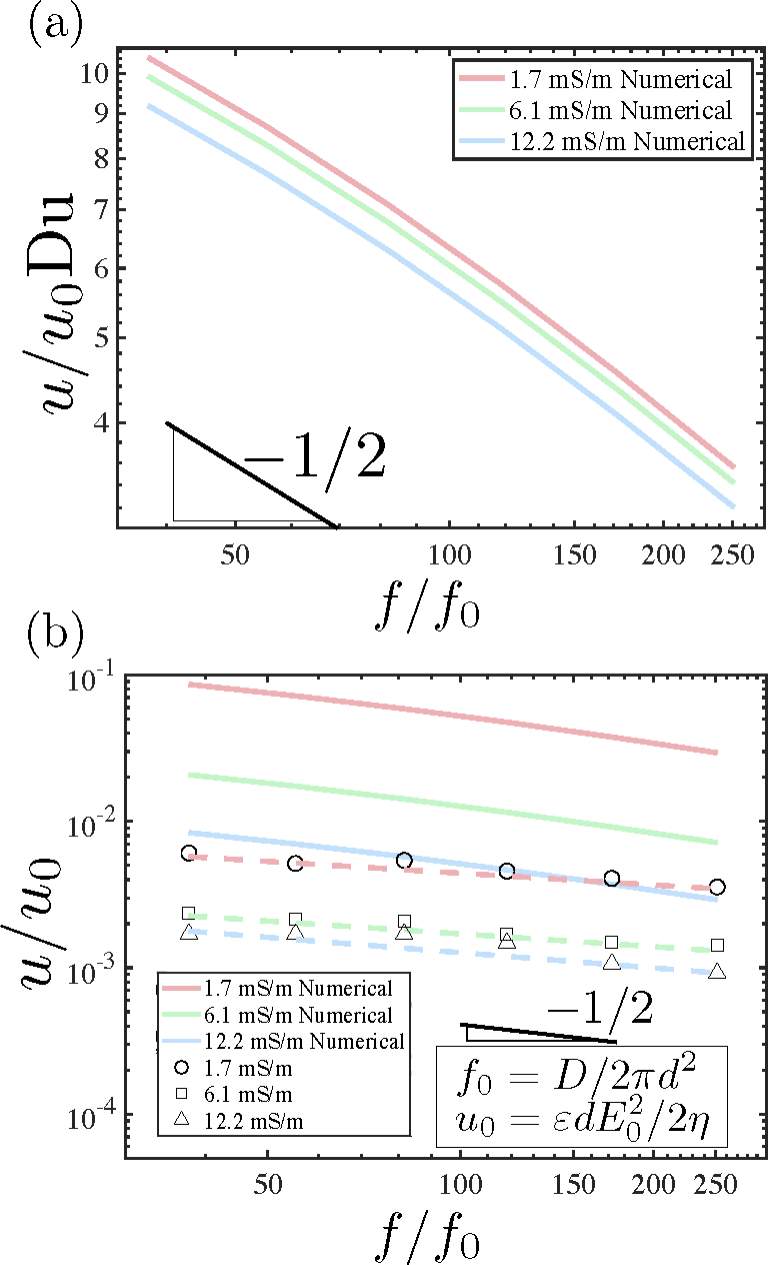
(a) Results of simulations for the measured zeta-potential of PDMS without surface treatment. (b) Comparison between experimental and simulation data. Du is determined as Du = *K*_s_/*σd*, with a typical surface conductance of *K*_s_ = 1 nS. The results of the simulations have been reduced to account for the effect of Pluronic on electroosmotic mobility.

The finite element software Comsol Multiphysics was used to solve the above equations in the domain of Figure 7. Geometrical dimensions are scaled with constriction width *d*, and angular frequencies are scaled with the reciprocal of the diffusion time associated with *d*, *d*^2^/*D* (frequencies are scaled with *D*/2*πd*^2^). The electric potential is scaled with ϕ_ther_, and the scale for fluid velocities results in *u*_0_ = *εdE*_0_^2^/2η. Figure 3(b) shows the solution of the fluid velocity field near the constriction for *f* = 65 Hz. The figure shows one of the four flow vortices generated by the slip velocity on the channel wall. The arrow field indicates the velocity direction, which gives rise to a circulating flow vortex as observed experimentally. The surface plot shows the velocity magnitude, which reaches a maximum near the tip of the constriction. To compare with experiments, the mean velocity magnitude was calculated within the boundary indicated in Figure 7, i.e., the area where the experimental velocity magnitude was measured. Figure 8(a) shows numerical results for the mean velocity magnitude scaled with *u*_0_Du as a function of frequency determined for three values of the zeta-potential of PDMS: −89.0 ± 1.2 mV for 1.7 mS/m, −83.2 mV for 6.1 mS/m, and −74.3 mV for 12.2 mS/m. These zeta-potential values were determined experimentally for a PDMS channel using the current-monitoring method, first reported by Huang et al.^30^ The velocity approximately decays with the square root of the frequency, as expected.

Figure 8(b) also shows experimental measurements of the velocity from Figure 4 scaled with *u*_0_Du. For each conductivity, Du is estimated from Du = *K*_s_/*σd* with *K*_s_ = 1 nS, a widely used value for the surface conductance and a value that was used in our previous work on insulating PDMS posts.^13^ According to Viefhues et al.,^31^ the addition of Pluronic reduces the electroosmotic velocity. We have used the current-monitoring method for measuring electroosmotic mobility of PDMS surfaces primed with Pluronic and we have observed a mobility reduction factor around 4. Specifically, the theoretical values in Figure 8(b) correspond to the data in Figure 8(a) divided by the following factors: 3.5 for 1.7 mS/m, 3.9 for 6.1 mS/m, and 4.5 for 12.2 mS/m, to take into account the reduction of surface mobility. In general, the theoretical results systematically overpredict the measured velocities by a factor ranging between 2 and 10. Three possible explanations for this discrepancy include the following: (1) The actual value for *K*_s_ is smaller than assumed; a least-square fit to the data gave a value of *K*_s_ = 0.16 nS. The Bikerman equation^25^ for the surface conductance in the diffuse layer predicts a value of *K*_s_ = 0.29 nS for our experimental parameters, closer to the result of our fitting. However, this result does not necessarily reflect that the Bikerman model for a bare surface describes our experimental situation, which corresponds to a surface treated with Pluronic. (2) A linear model for CPEO flows was used, which is valid as long as the amplitude of the electric field remains small, *E*_0_*d* ≲ *k*_B_*T*/*e*. This assumption might not be satisfied within the constriction, where the electric field is high. (3) The model is valid for a small Du, which means that surface conduction is much smaller than in the bulk. This might not be the case at the tips of the constrictions.

### Analysis of the Influence of Electrothermal Flows and Induced-Charge Electroosmosis

Electrical currents produce Joule heating within electrolytes, and this can lead to gradients in temperature that create nonhomogeneous regions of conductivity and permittivity in the liquid. The electric field acting on these conductivity and permittivity gradients gives rise to a bulk fluid motion known as electrothermal flow.^16,32^ Electrothermal flows in iDEP constrictions were first reported using high conductivity phosphate buffer solutions.^33^ Wang et al.^17^ reported electrothermal flow in constrictions using 10 mM KCl and AC fields with a frequency of 1  kHz. Also, electrothermal flows were used to enrich submicron particles suspended in PBS.^34^ However, electrothermal flows do not play a role in our experiments because the electrolyte conductivity is not high enough for Joule heating to produce significant changes in temperature. In addition, the effect of electrothermal flows is found for frequencies of the order of the reciprocal of the charge relaxation time of the electrolyte (around hundreds of kHz), while the flows studied here vanish for frequencies much larger than *D*/(2*πd*^2^) (around 1 Hz for our experimental conditions).

Recent works have discussed the appearance of induced-charge electroosmosis (ICEO^35^) within microfluidic constrictions^17^ and corners.^27^ ICEO flows typically occur on metal surfaces in contact with electrolytes subjected to DC or AC electric fields, and its origin is the interaction of the field with the electrical charges induced at the metal–electrolyte interface. The most important difference between the mechanisms for ICEO and CPEO flows is that, in the latter case, the surface charge is not modified by the applied electric field. ICEO theory for insulating objects^36,37^ predicts a slip velocity that decays around frequencies of the order of the reciprocal of the charge relaxation time of the electrolyte (σ/2*πε*, ≈0.3–3 MHz for our experimental parameters). This frequency is orders of magnitude higher than the typical frequency in our experiments (below 10 kHz). Additionally, it can be shown that ICEO velocities on insulating walls are negligibly small compared to CPEO.^13^

## Conclusions

We have experimentally demonstrated the presence of quadrupolar fluid flows induced by AC electric fields around constrictions in microfluidic channels for low conductivity electrolytes. The flow pattern and magnitude was determined using fluorescent tracer beads (500 nm diameter). The flow patterns are visible for frequencies above 10 Hz because particle electrophoresis dominates for lower frequencies. The magnitude of the fluid velocity decreases with the conductivity of the electrolyte and approximately scales with the reciprocal of the square root of the frequency of the AC field. Also, the fluid velocity vanishes for frequencies much higher than the reciprocal of the electrolyte concentration diffusion time (*D*/*d*^2^, with *D* the diffusion coefficient of the electrolyte and *d* a typical length, for example, the constriction width). Particle dielectrophoresis occurs for frequencies higher than the latter. Significantly, the height of the channel has a major influence on the particle behavior and fluid patterns. Specifically, for shallow channels (10 μm high), trapping of the particles occurs on both sides of the constrictions. Further work is needed to clarify the mechanism responsible for this trapping. In comparison with the tall channels, the weakening of the CPEO flows may be due to the proximity of the top and bottom walls. Surface conduction leads to concentration polarization and a rectified electric field that probably persist in these shallow channels. Therefore, these phenomena should be taken into account in the theoretical explanation of the trapping, which is clearly different from classical DEP. Previous work on iDEP devices describes particle behavior as a competition between DEP forces, that scale with the square of the electric field, and the particle motion arising by the combined effect of electrophoresis and liquid electroosmosis, that scale linearly with the electric field. However, large discrepancies between theory and experimental data have been reported.^38^ Given the strength and structure of these quadrupolar flows, they must be considered as an additional mechanism affecting the force balance on particles, especially at low frequencies and conductivities. For example, trapping by pDEP will be distorted by these flows (as observed for the 500 nm particle which are only trapped when the flows vanish). Also, particles experiencing nDEP could be subjected to an apparent larger DEP force because flow recirculation at the constriction moves the particles away from it. Future work should focus on the influence of these flows and trapping regime to improve the understanding and application of iDEP and other techniques such as DLD.

The experimental trends for the fluid flows are in agreement with the rectified electroosmosis that arises from the concentration–polarization due to surface conductance on the channel walls,^13^ a mechanism that we named concentration–polarization electroosmosis (CPEO). We compared the experimental data with the predictions of an electrokinetic model based on the approximations of a small Du number and amplitude of electric fields. The results of the model qualitatively agree with observed trends, although the velocities are systematically overestimated if a typical value of 1 nS is assumed for the surface conductance. The assumptions of a small Du and *E*_0_ might not be correct for this system, and more theoretical and experimental characterization is required along with an accurate determination of the surface conductance of these surfaces.
